# Quick Decline and Stem Pitting *Citrus tristeza virus* Isolates Induce a Distinct Metabolomic Profile and Antioxidant Enzyme Activity in the Phloem Sap of Two Citrus Species

**DOI:** 10.3390/plants12061394

**Published:** 2023-03-21

**Authors:** Susana A. Dandlen, José P. Da Silva, Maria Graça Miguel, Amílcar Duarte, Deborah M. Power, Natália Tomás Marques

**Affiliations:** 1 MED—Instituto Mediterrâneo para a Agricultura, Ambiente e Desenvolvimento, Faculdade de Ciências e Tecnologia, Universidade do Algarve, Campus de Gambelas, 8005-139 Faro, Portugal; 2 Centre of Marine Sciences (CCMAR/CIMAR LA), Universidade do Algarve, Campus de Gambelas, 8005-139 Faro, Portugal; 3 CEOT—Centro de Eletrónica, Optoeletrónica e Telecomunicações, Faculdade de Ciências e Tecnologia, Edif. 8, Universidade do Algarve, Campus de Gambelas, 8005-139 Faro, Portugal

**Keywords:** antioxidant enzymes, flavonoids, LC-HRMS2, metabolomics, phloem sap, plant-virus interaction, sieve element

## Abstract

Susceptibility to the severe *Citrus tristeza virus* (CTV), T36, is higher for *Citrus macrophylla* (CM) than for *C. aurantium* (CA). How host-virus interactions are reflected in host physiology is largely unknown. In this study, the profile of metabolites and the antioxidant activity in the phloem sap of healthy and infected CA and CM plants were evaluated. The phloem sap of quick decline (T36) and stem pitting (T318A) infected citrus, and control plants was collected by centrifugation, and the enzymes and metabolites analyzed. The activity of the antioxidant enzymes, superoxide dismutase (SOD) and catalase (CAT), in infected plants increased significantly in CM and decreased in CA, compared to the healthy controls. Using LC-HRMS^2^ a metabolic profile rich in secondary metabolites was assigned to healthy CA, compared to healthy CM. CTV infection of CA caused a drastic reduction in secondary metabolites, but not in CM. In conclusion, CA and CM have a different response to severe CTV isolates and we propose that the low susceptibility of CA to T36 may be related to the interaction of the virus with the host’s metabolism, which reduces significantly the synthesis of flavonoids and antioxidant enzyme activity.

## 1. Introduction

Citrus (*Citrus* spp.) genus is an economically important fruit crop worldwide. The sustainability of citrus production is threatened by the *Citrus tristeza virus* (CTV), which is a ssRNA (+) virus and a member of the *Closteroviridae*. The virus is transmitted by aphid species and by man through the propagation of infected budwood [1] and affects primarily the phloem-associated cells, although it can also replicate in the immature xylem tracheid cells [2]. The devastating consequences of the virus on citrus plants led to the virus being classified as a quarantine pest [1].

CTV elicits three distinct disease syndromes in *Citrus* spp., tristeza disease, stem pitting and seedling yellows. The development of the syndromes depends on the infectivity of the virus isolate, the infected citrus species and the scion-rootstock combination [3]. The spread of tristeza disease has been related to the use of susceptible *Citrus aurantium* (sour orange, CA) as a rootstock, and occurs only when the grafted scions are sweet orange (*C. sinensis* (L.) Osbeck), mandarin (*C. reticulata* Blanco) or grapefruit (*C.* × *paradise* Macf.). These rootstock-scion combinations, when infected by a quick decline isolate, display the tristeza disease that can lead to the death of the plants, a problem that was circumvented through replacing CA by citrus rootstock tolerant to CTV, although without similar agronomic characteristics.

A successful infection depends on the virus’s ability to multiply, that is to establish interactions with plant proteins and to suppress the host plants defense response, which determines the degree of susceptibility of different citrus species to CTV [4]. CTV uses mainly the sieve elements to circulate throughout the plant and infect distal plant parts but to replicate it needs to enter the phloem companion cells in a cell-to-cell movement. Plant susceptibility to CTV was proposed to be related to the virus’s ability to exit the sieve elements and enter the adjacent phloem cells where it replicates [4], to activation of the salicylic acid (SA) defense pathway in the host *Citrus* spp. and to the activity of the viral silencing suppressors p20 and p23 [5].

Alemow (*Citrus macrophylla* Wester, CM) and CA have different susceptibility to CTV. CM is highly susceptible to the clone CTV_T36-GFP that carries a quick-decline isolate and promotes the infection of clusters of companion cells in the bark slip tissue, whereas in CA it induces the infection of single cells [4]. According to the authors, the reduced number of CTV_T36-GFP infected cells in CA was related to a low permissiveness to virus progression, which led to the designation of CA as having low susceptibility to the virus.

As for the infected plant, a viral infection by symptomless or severe CTV isolates impairs numerous metabolic and physiological processes in citrus plants [6]. In response to an infection by a virus, plants often produce reactive oxygen species (ROS), which trigger plant defense mechanisms including systemic acquired resistance (SAR) that develops systemically to distal and uninfected parts of the plant [7,8]. Production of SA and nitric oxide (NO) by plants in response to a virus can decrease or suppress the ROS detoxifying systems, and thus favor programmed cell death and block disease dissemination [9]. In addition to its beneficial actions, excessive formation of ROS can damage plant structures [10]. One of the mechanisms that controls ROS levels is the enzymatic antioxidant system that includes superoxide dismutase (SOD), catalase (CAT), guiacol peroxidase (POD), polyphenol oxidase (PPO), ascorbate peroxidase (APX), monodehydroascorbate reductase (MDHAR), dehydroascorbate reductase (DHAR), glutathione reductase (GR), glutathione peroxidase (GPX) and glutathione *S*-transferase (GST) [10,11]. Plants also have non-enzymatic antioxidant compounds (e.g., water-soluble ascorbate, reduced glutathione, phenolic compounds and lipid-soluble carotenoids and tocopherols) which act as radical scavengers that protect the plant from the damaging effects of biotic stresses [12]. 

CTV infection increases antioxidant enzyme activity (e.g., SOD and APX) and increases ROS scavenging activity in the young stem branches of sweet orange ‘Westin’ [6] and in the new shoots of Mexican lime (*C. aurantifolia*) [13]. However, in Mexican lime leaves, infection by CTV decreased the activity of SOD, and this was taken to suggest that this enzyme has an active role in defense mechanisms against ROS as its presence constitutes one of the first defenses [10]. The phloem exudates evaluated in pumpkin and cucumber displayed SOD, APX and DHAR activities as well as proteins controlling the redox balance during oxidative stress [11,14], but there is a lack of information regarding the activity of these enzymes in the phloem sap of perennials. 

The phloem sap has been identified as a pathway for the rapid transport of NO, ROS such as hydrogen peroxide (H_2_O_2_) and SAR metabolites resulting from the response of the host to a viral infection [12,15,16]. Recent research has revealed that phloem cells not only transport but also synthesize signalling molecules, small RNAs, hormones and proteins, some of which are produced to reprogram plasmodesmata (PD) permeability and limit virus progression [16,17,18,19]. The phloem sap in sieve elements is thus an important location for ROS detoxification [11]. During a virus infection, significant transcriptional and translational changes in phloem tissues are induced compared to surrounding non-phloem tissues [17]. So, the phloem is a site of mediation and control of host responses during viral infection where specific virus-host interactions take place that affect the host defense responses [17]. 

Upon infection by CTV, gene transcripts related to plant defense were modified in infected plants [20] with changes in primary and secondary metabolite production as part of the immune response [21]. As an example, *C. sinensis* controls Huanglongbing disease with formation of secondary metabolites (mainly phenolic acids), both in the leaves [22] and phloem sap [21]. *Citrus* spp. naturally accumulate a great number of secondary metabolites, among which the phenolic compounds (terpenoids, phenylpropanoids and flavonoids) are differentially expressed in distinct plant tissues [23]. As CTV circulates mainly in the sieve elements, it is expected that the alterations induced by the presence of the viral pathogen are reflected and detectable in the composition of the phloem sap. Previous work reported an increase in amino acids, organic acids (oxalic, malic, and citric acids) and sugar acids, and a decrease in glucose and sugar alcohols in the phloem sap of CTV-infected plants [24]. Unfortunately, there is little information about the metabolic alterations of phloem parenchyma cells and sieve elements in CTV-infected citrus [24] and much more work is required to explore these changes. 

The main goal of this study was to establish the defense response of CA and CM to the CTV quick decline isolate T36 by characterizing the activity of antioxidant enzymes and phloem sap metabolites upon viral infection. To achieve this end, a comparison was made with CA and CM infected with the severe stem pitting isolate T318A. Since these two citrus species display distinct susceptibilities and symptoms to the isolates T36 [3] and T318A [25], metabolite profiles that distinguish susceptibility to the virus were procured. To this end, phloem sap was collected by centrifugation and analyzed to detect the enzymatic activity of SOD, CAT, APX and DHAR as well as the metabolite profile of the phloem sap of CA and CM infected by the two severe CTV isolates. 

## 2. Results and Discussion

### 2.1. Enzymatic Antioxidant Profiling

**Figure 1:**
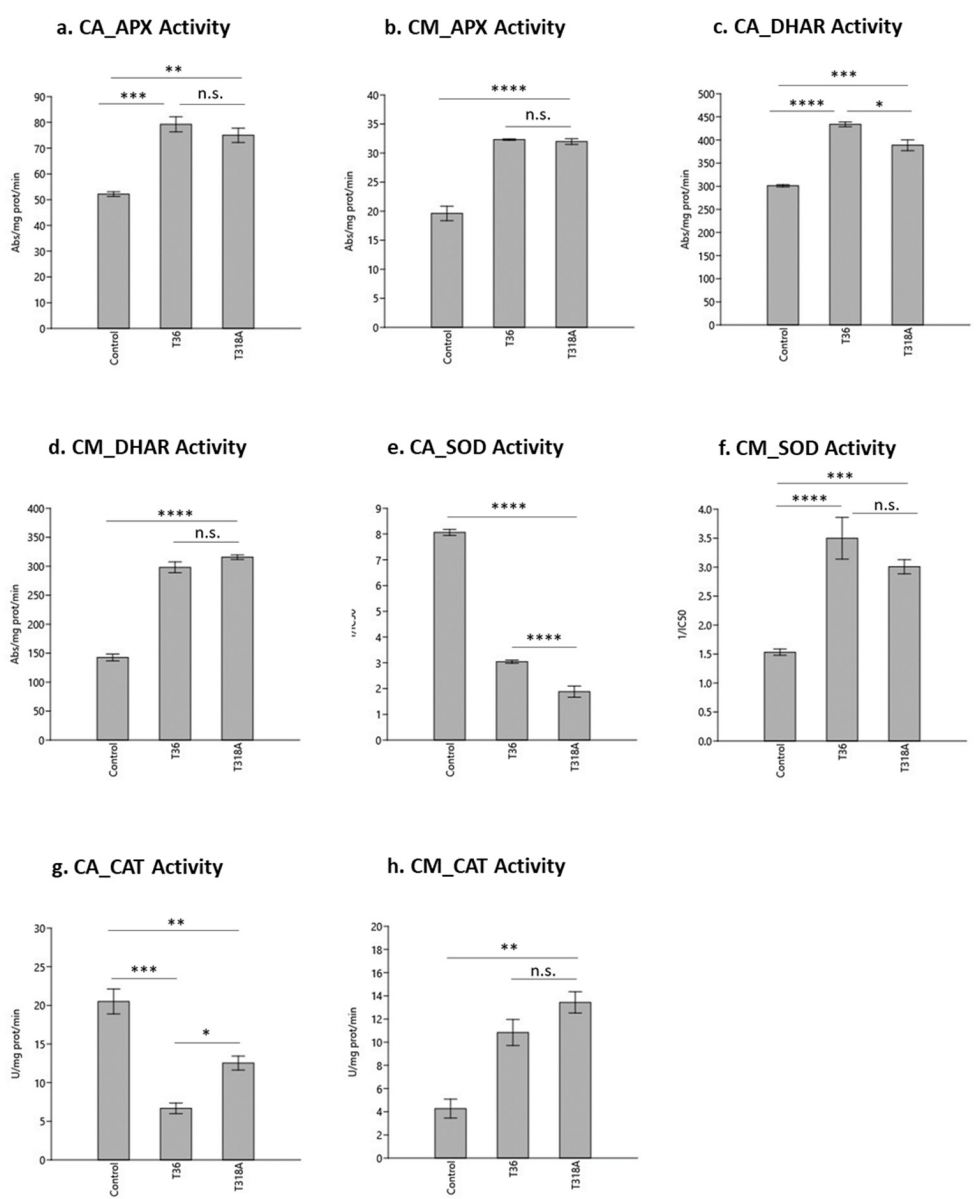
Activity of antioxidant enzymes in the phloem sap of sour orange and alemow, healthy and infected with CTV T36 isolate or T318A. Comparisons were made between control and infected samples and between the infected samples. (**a**) ascorbate peroxidase (APX) activity in CA; (**b**) APX activity in CM; (**c**) dehydroascorbate reductase (DHAR) activity in CA; (**d**) DHAR activity in CM; (**e**) superoxide dismutase (SOD) activity in CA 1/IC50; (**f**) SOD activity in CM 1/IC50; (**g**) catalase (CAT) activity in CA; (**h**) CAT activity in CM. ****: *p* < 0.0001; ***: *p* < 0.001; **: *p* < 0.01; *: *p* < 0.05; n.s.: not significant (*p* > 0.05).

The activity of APX, DHAR, SOD and CAT, involved in detoxification of ROS, was evaluated in the phloem sap of bark samples. APX reduces hydrogen peroxide to water and dehydroascorbate, using ascorbic acid as the reducing agent [26]. A significant increase in APX activity occurred in the phloem sap of infected plants of CA and CM (*p* < 0.001 and *p* < 0.0001, respectively), compared to the controls (Figure 1a,b). This is indicative of a strong stress condition in both CA and CM plants, although the type of virus isolates did not cause a significant difference in the response. Furthermore, the APX activity in healthy and infected plants was higher in CA than in CM. In a previous study APX was also increased in bark tissue of a CTV infected sweet orange, grafted on CA [27].

In the reduction of hydrogen peroxide by APX, ascorbate (ASC) is converted to dehydroascorbate (DHA) and then recovered by the activity of monodehydroascorbate reductase (MDAR) and dehydroascorbate reductase (DHAR) [9]. In this process DHAR reduces DHA to a short-lived compound, using glutathione (GSH) as an electron donor [28]. The DHAR enzyme, involved in the ascorbate–glutathione (ASC–GSH) cycle, was more active in the phloem sap of infected plants than in the control of both CA and CM, although the activity was significantly higher in CA (*p* < 0.001). The DHAR activity was significantly higher in CA plants infected by isolate T36 than by isolate T318A (*p* < 0.0001) (Figure 1c). Differences between the DHAR activity in phloem sap of infected and non-infected CM were also significant (*p* < 0.0001) but the 2 CTV isolates did not elicit a significantly different response (Figure 1d). 

SOD catalyzes the dismutation of superoxide radical anion into molecular oxygen and hydrogen peroxide [26]. SOD was significantly reduced in the phloem sap of CA infected with CTV compared to the control (*p* < 0.0001) (Figure 1e). In addition, the SOD activity was significantly higher in CA infected with the isolate T36 than with isolate T318A (*p* < 0.0001). The SOD activity in the phloem sap of healthy CM plants was significantly lower than in the infected plants (*p* < 0.001) (Figure 1f), and no significant difference in activity occurred between plants infected with the T36 or T318A isolates (Figure 1f). Previous studies have reported reduced SOD activity in *Phaseolus vulgaris* infected with *White clover mosaic potexvirus* (WCIMV) [29] and in a resistant soybean cultivar infected with *Soybean mosaic virus* [30]. However, SOD activity was high in stem bark samples of *C. sinensis* ‘Westin’ infected with CTV, although no information was reported about the severity of the CTV isolate [6]. In the present study, by assessing the activity of SOD in CA and CM, the enzyme activity in response to CTV was genotype-dependent (Figure 1e,f). Similarly, in the ‘Tarocco’ sweet orange grafted onto the tolerant rootstock Carrizo citrange, SOD activity was increased compared to the same scion grafted onto CA [27]. In contrast, in the highly susceptible Mexican lime, SOD activity was suppressed when the plant was infected by CTV [10].

CAT, which converts hydrogen peroxide into water, was reduced in two *Prunus armenica* L. cultivars after infection with *Plum pox virus* (PPV) [31]. CAT was also inhibited in *Arabidopsis* infected with *Cucumber mosaic virus* (CMV), and this was explained by the direct interaction of isoenzymecatalase 3 (CAT3) with CMV 2b protein [32]. The same signature occurred in healthy CA plants, and CAT activity in the phloem sap was significantly higher in healthy compared to CTV infected plants (*p* < 0.001) (Figure 1g). In CA infected by the isolate T318A, CAT activity was twice as high (*p* < 0.05) as that recorded when infected with isolate T36 (Figure 1g). In contrast, the CAT activity increased significantly in CM plants infected with CTV (*p* < 0.01), and no significant difference was identified between plants infected by T36 and T318A (Figure 1h). 

The activity of antioxidant enzymes keeps ROS at optimal levels. The decrease in antioxidant enzyme activity, particularly SOD and CAT in CA infected plants may be a defense response of the plant to the viral infection since it leads to increased superoxide anion radicals and hydrogen peroxide. These enzymes may be biomarkers that explain the low susceptibility of CA to CTV. The increase in superoxide and hydrogen peroxide due to the diminished activity of SOD and CAT can be compensated by the increase of APX and DHAR activities, which convert hydrogen peroxide to water and recover ascorbic acid through the action of DHAR. The increased activity of APX and DHAR has previously been related to the physiological changes triggered by cellular damage caused by virus replication [10,29]. Another possible explanation for the significantly lower activity of SOD and CAT may be assigned to interference by the virus. 

Other mechanisms that can occur in plants faced with a biotic stress, as reported by [33,34], include the induction of nonenzymatic antioxidants (ASC and GSH) or other metabolites such as tocopherol, flavonoids, alkaloids, and carotenoids [9]. In CM infected plants, the activities of all tested antioxidant enzymes increased, which presumably leads to a decrease in ROS. 

### 2.2. Phloem Metabolites

The metabolites of the phloem sap of infected and non-infected plants of both citrus species was processed using Compound Discoverer 3.3 by running an untargeted metabolomics workflow to detect and annotate [35] the differences between infected and non-infected plants. The metabolite profiles of non-infected plants of both species were first compared using the same approach. Table 1 presents a list of compounds annotated in the phloem sap of CA and CM control plants.

The metabolite composition of the phloem sap of non-infected plants was significantly different in the two citrus species CA and CM (Table 1). Flavonoids, namely flavonols and their derivatives such as quercetin, isorhamnetin and biorobin; flavones and their derivatives such as apigenin, luteolin and rhoifolin; and flavanones and their derivatives such as naringenin, naringin and hesperetin, are significantly more abundant in CA. The only annotated flavonoid compounds showing higher content in CM were rutin and eriocitrin. In contrast, phenolic acids such as 4-coumaric acid, dihydrocaffeic acid and 2-hydroxycinnamic acid had a significantly higher content in CM (Table 1). The main flavonoid derivatives in CA phloem sap were glycosylated and/or methoxylated compounds. Polymethoxyflavones such as gardenin D (5,3′-dihydroxy-6,7,8,4′-tetramethoxyflavone), casticin (5,3′-dihydroxy-3,6,7,4′-tetramethoxyflavone), eupatilin (5,7-ihydroxy-6,3′,4′-trimethoxyflavone) and nobiletin (5,6,7,8,3′,4′-hexamethoxyflavone) have previously been reported in fruits of CA and other citrus species [36,37].

Most compounds annotated in the phloem sap of CA and CM (Table 1) have previously been reported in different matrices of citrus species and have been used as a tool for taxonomy to distinguish citrus species and to identify plant parts [23,38]. For example, CA flowers are a rich source of phenolic acids and flavonoids, including polymethoxy flavonoids (PMFs) [39]. PMFs, glycosylated or not, have been reported in several citrus species, namely in the fruit peel of sweet orange, but also in seeds and juice, in stems and leaves [38,40,41]. Neohesperidin is the compound responsible for the typical bitterness of fruits and juices from CA [42], and the flavanones hesperetin and naringenin, and the glycosides neohesperidin and naringin, as well as the flavone apigenin, are chemical markers of CA fruit peels [36]. Major roles of flavonoids in plants include modulation of ROS production [43]. In fact, the formation of ferulic acid, a potent free-radical scavenger and antioxidant, was induced in citrus leaves of several species after treatment with SA [44]. 

Information on the nature and role of the phenolic compounds present in the phloem sap of citrus species is scarce. In fact, phenolic compounds were described as not being very abundant in the phloem sap of sweet orange ‘Valencia’ and orange jasmine, where ferulic acid was found at low levels [19]. This is in line with our results for healthy CM as compared to healthy CA. Thus, a characteristic of healthy CA plants but not CM plants was the relatively high levels of flavonoids in the phloem sap. As far as we know, this is the first time that compounds such as flavanones naringin, hesperidin and neohesperidin were detected in significant amount in the phloem sap of a citrus species. Of interest is a proteome study of the phloem sap in *C. sinensis* (L.) Osbeck which revealed that specific proteins were associated with tissues that surround the vasculature [45]. This raises the possibility of contamination of samples with compounds that do not circulate in the phloem. Irrespective of this, the rich composition of phenolic compounds in the phloem sap of CA plants is very distinct from CM.

Table 2 lists the compounds significantly upregulated or downregulated in the phloem sap of CA and CM upon infection with T36 and T318A. For an easier match of compounds common to Table 1 and Table 2, these have been marked with a symbol (^♦^) preceding its name. In the case of CM, compounds such as 5,6′-dihydroxy-6,7,8,2′-tetramethoxyflavone and isovanillic acid were significantly higher in the infected phloem sap while others such as 4-aminobenzoic acid (PABA) and isoferulic acid were significantly lower. PABA was found to be essential for enhancing resistance to *Xanthomonas axonopodis* and *Cucumber mosaic virus* in pepper [46] raising interesting questions about the effect of the significant reduction of PABA in CM (Table 2). As seen in Table 1, the polymethoxylated flavonoid 5,3′-dihydroxy-3,6,7,4′ tetramethoxyflavone was present in significantly higher amounts in CA than in CM. The infection by CTV significantly affected the secondary metabolites in CA and the polymethoxylated flavonoid was much higher in plants infected by T318A compared to T36. This was not the case for CM where polymethoxylated flavonoid 5,6′-dihydroxy-6,7,8,2′-tetramethoxyflavone was found in significant amounts upon infection. These results reveal that CM and CA have a different response to CTV and we propose polymethoxylated flavonoids may influence infection susceptibility. Independent of the polymethoxylated flavonoids produced by infected CA and CM, they may be coupled to ascorbate regeneration via the DHAR and glutathione reductase system, which acts as an H_2_O_2_ scavenging mechanism [47].

The significantly lower amount of isoferulic acid in CM (Table 2) may be due to the significantly increased biosynthesis of isovanillic acid, which is obtained from isoferulic acid by degradation of the side chain C_3_, through β-oxidation as occurs in fatty acid metabolism. This process may represent an important energy source for plant defenses since the total oxidation of the compound generates high ATP equivalents by producing one acetyl-CoA, one NADH and one FADH_2_ [48]. Isovanillic acid was also significantly higher in CM infected with T36, but not with T318A (Table 2). In addition, both CM and CA infected by T36, had lower amounts of isoferulic acid, which has radical scavenging activity [47], and this is in line with the decrease of flavonoids in CA. These results show the specific and distinct responses of CM to two distinct severe viral isolates.

The infection by T318A caused a different metabolite profile in CA compared to CM with fumaric acid and *cis*-aconitic acid being in significant amounts in CA upon infection.

In the case of CM, the defense response to the virus T36 involved the lignan 2-[[6-hydroxy-4-(4-hydroxy-3,5-dimethoxyphenyl)-3-(hydroxymethyl)-5,7-dimethoxy-1,2,3,4-tetrahydronaphthalen-2-yl]methoxy]-6-(hydroxymethyl)oxane-3,4,5-triol and the flavonoid quercetin (Table 2), a compound known to induce bacterial and fungal pathogen resistance [49]. Formation of lignan by oxidative coupling of *p*-hydroxycinnamic acid radicals catalyzed by peroxidases could be an oxidation pathway from hydrogen peroxide without water formation [50]. Such secondary metabolites generally exhibit antimicrobial activity. In addition, oxidative coupling of *p*-hydroxycinnamic acid alcohol radicals catalyzed by peroxidases may lead to lignification, a structural barrier restricted to vascular tissues, especially xylem, that is important for combating some pathogens [50]. In common with the response of CM to T381A, there was also increased formation of SA and pipecolic acid in CA (Table 2), which are part of the SAR mechanism that has a vital role in plant defense [51]. SA in the presence of peroxidases could produce superoxide anion radicals even in the absence of hydrogen peroxide, as a response to pathogen attacks [52]. Pipecolic acid could be important in the modulation of cellular redox homeostasis, as previously observed for tomato plants under abiotic stress [53]. In CM there was also a significant increase in the contents of 3-[2-(β-D-Glucopyranosyloxy)-4-methoxyphenyl] propanoic acid (Table 2). A massive accumulation of pipecolic acid and SA was found in tobacco plants infected with TMV and CMV after signal perception and manifestation of SAR in distant leaves [16]. The low susceptibility of CA to CTV suggests partial resistance to the virus and was attributed to the presence of SA [5], which was not detected in CA in the present study possibly due to the amount of flavonoids and other compounds in the phloem sap that reduce stress sensitivity.

A glycosylated ferulic acid derivative was significantly increased in CM as a response to the infection (Table 2) and the increased water-solubility conferred by glycosylation may make transport in the phloem sap and thus throughout the plant easier [54]. Based on the metabolome results, CM had a different response to T36 and T381A, and significant differences in several metabolites were annotated (Table 2). Overall, the response of CA and CM to the presence of each severe isolate was different, with the induction and transport of different metabolites in the phloem sap.

The increase in the phloem sap of TCA organic acids (fumaric acid and *cis*-aconitic acid) in CA and CM infected by T318A and T36, respectively, is a common feature of plants infected by viruses and is linked to the energy needs for virus multiplication and plant defense [43]. TCA also play an important role as regulator of flavonoid biosynthesis and intermediate metabolites of this cycle were previously reported in the phloem sap of *C. sinensis* cv. ‘Valencia Late’ [19]. 

The role of flavonoid compounds produced in plants is that of a general defense response against pathogens and they are involved in plant immunity as well [49,55]. Flavonoids promote the biosynthesis and accumulation of SA and the activity of mitogen-activated protein kinases (MAPK) [56] and rutin confers greater resistance of *Arabidopsis* to bacteria through the induced expression of several genes related to pathogenesis [49]. Enhanced production of flavonoids in transgenic herbaceous and perennial plants confers resistance to several fungi species [57]. Despite knowledge accumulated on how flavonoids operate as antimicrobial compounds [49,56,58], the molecular mechanism underlying their role in the defense response to viruses has not been extensively studied. Similarly, a low accumulation of flavonoids in transgenic medicago and barley increased the susceptibility to infection by pathogenic fungi [57]. We propose that flavonoid compounds may also influence citrus species susceptibility to CTV. 

The highly susceptible Mexican lime produces less phenolic compounds in the leaves when infected by CTV [10]. Analogously, in the present study, the high CTV-susceptible CM had significantly less diverse and modified secondary metabolites in the phloem sap compared to the more resistant CA. The absence of a notorious T36 spread in the CA sieve elements [25] has previously been correlated with phenolic SA, which is part of the SAR response; when reduced, it is associated with an enhanced spread and accumulation of T36 [5]. Thus, the high concentration and diversity of phenolic compounds in the phloem sap of healthy CA (Table 1), may explain its lower susceptibility to CTV.

Since an increase in flavonoid production is expected as a general plant response to the presence of a pathogen, the significant decrease in these compounds in CA phloem sap after T36 infection compared to healthy control plants (Table 2) was unexpected. Our hypothesis is that flavonoid biosynthesis may be blocked by the virus. Future work needs to be developed to understand better the role of the phenolic compounds in CTV infection and how they interfere in CA response. In addition to flavonoids, healthy CA had a higher antioxidant enzyme activity in the phloem sap, compared to healthy CM. A significant correlation between high antioxidant activities and phenolic contents extracted from citrus peels has previously been described [59]. We propose that the differences in phloem sap metabolites in healthy CA and CM may contribute to explain their differing responsiveness to CTV. 

The CTV isolates used in this study generate distinct syndromes in CA and CM. The quick decline isolate T36 can lead to the death of citrus plants, but it only affects specific hosts when they are grafted onto the CA rootstock. T36 infection of CM causes only very mild stem-pitting symptoms [60]. Results from our study reinforce previous reports that different severe isolates of CTV induce different syndromes in the same citrus species [25]. CTV isolates employ distinct strategies to interact with different citrus hosts. A known interplay is the interaction of citrus hosts with viral p33, p18 and p13 gene products [4]. Viral proteins p20 and p23 also play a role by interfering with the SA signaling defense, with a high suppressor activity of the plant RNA silencing mechanism for the most virulent isolates [25]. In the present study, the most severe isolate T36 had a high impact on CA metabolism compared to T318A, and significantly reduced flavonoids, including those that were polymethoxylated, as well as SOD and CAT antioxidant enzyme activities. The results of the present study suggest that the phloem sap metabolites, particularly the flavonoid compounds, may have a role in citrus susceptibility to CTV and in the progression of the virus in the conducting vessels and its ability to infect the plant. As CTV isolates employ distinct strategies to interact with different citrus hosts [25], it is conceivable that plant resistance mechanisms may involve metabolites already available and/or produced in the phloem vascular system.

## 3. Materials and Methods

### 3.1. Plant Material and Virus Isolates

Twelve four-year-old plants of each *Citrus aurantium* L. (CA) and *C. macrophylla* Wester (CM) species were grown in an artificial potting 50:50 mix of pine bark and coconut fibre and kept in an insect-proof greenhouse. Plants were maintained under ambient conditions (temperature, relative humidity and illumination) for the Algarve, Portugal, during 2016 to 2020 (RNA extraction was performed in March 2020). Four plants of each species CA and CM were graft-inoculated with the CTV severe stem pitting isolate T318A [61] and another four plants were inoculated with the quick-decline T36 isolate, kindly provided by Dr. Leandro Peña (Institute for Plant Molecular and Cell Biology—Polytechnic University of Valencia). CTV graft-inoculation was performed with two inoculum young stem pieces from one T318A-infected and one T36-infected *Citrus macrophylla* plant. Four non-infected healthy plants of each species were used as controls. All citrus plants were kept under controlled conditions of watering, and phloem sap extraction was performed one year after CTV inoculation. 

### 3.2. Total RNA Extraction and CTV Detection by Reverse Transcription-PCR (RT-PCR)

Host CTV infection was confirmed by RNA extraction followed by an RT-PCR. One randomly sampled young shoot was collected from each plant to confirm the presence of CTV in infected plants or its absence in healthy controls. Detached slices from young bark tissue (herbaceous material, 100 mg) of control or infected plant twigs were used. The detached tissue constitutes the external layer of the young stems that separates the cambial layer between vascular tissues, with the extracted layer carrying the phloem tissue in its inside.

Plant material was macerated in liquid nitrogen and TRI Reagent (Sigma-Aldrich, St. Louis, MO, USA) was used for extraction of total RNA (totRNA) according to the manufacturer’s instructions. RNA was stored at −80 °C until use. RNA concentration was determined using a NanoDrop 1000 UV-Vis Spectrophotometer (Thermo Scientific, Waltham, MA, USA). RT-PCR mix was performed in a final volume of 25 μL as described previously [62], containing 0.625 U of Dream *Taq* DNA polymerase (Thermo Scientific, USA) and 100 ng of totRNA. Primer pairs used were CTV1 Fw (5′ ATGGACGACGARACAAAG 3′, with R = A/G) and CTV2 Rv (5′ TCAACGTGTGTTYAATTTCC 3′, with Y = C/T), to amplify the complete sequence (672 bp) of the p25 gene. totRNA from a healthy *C. macrophylla* was used as a negative control. For the positive control, the totRNA of a T318A CTV infected *C. macrophylla* was used. A T100 Thermal Cycler (BioRad, Hercules, CA, USA) was used with the following parameters: one step at 37 °C for 60 min, one cycle at 94 °C for 2 min followed by 35 cycles at 94 °C for 30 s, 52 °C for 40 s and 72 °C for 40 s, with an extension time of 72 °C for 5 min. The RT-PCR product was purified using a GFX™ PCR DNA and Gel Purification Kit (GE Healthcare, Chicago, IL, USA) following the manufacturer’s instructions. The purified RT-PCR product was sequenced in one direction using the CTV1 Fw primer and the identity confirmed searching against the NCBI database (http://blast.ncbi.nlm.nih.gov/Blast.cgi (accessed on 3 November 2022) using the blastx algorithm.

### 3.3. Phloem Sap Collection

Phloem sap was extracted from four biological samples of each citrus species CA and CM, infected with each CTV isolate, and the respective controls. The phloem sap was collected from young stems with around 0.5 cm diameter, in the month of March 2020, one-year post-inoculation of CTV. The collection of citrus phloem sap was made in accordance with [63,64]. Briefly, young stems were cut with 1.5 cm length and the external layer of the stem was detached, carrying phloem-associated cells on the inside of the excised bark. The external layer of the stems was rinsed in distilled water and dried with filter paper. Then several pieces of stem were placed vertically in a 0.5 mL eppendorf tube punctured with small holes at the base. Equal amounts of plant material, 100 mg, were placed in each eppendorf for phloem sap extraction. These tubes were inserted into a 2 mL collection tube, in order to collect the phloem sap during the centrifugation process at 4 °C and 13,800× *g* during 20 min. The obtained phloem sap was stored at −80 °C before use. Equal amounts of phloem sap obtained from single individuals were injected for metabolomic analysis (see Section 3.5).

### 3.4. Enzymatic Antioxidant Assays

Enzymatic antioxidant assays were performed to analyze the activity of superoxide dismutase (SOD), catalase (CAT), dehydroascorbate reductase (DHAR) and ascorbate peroxidase (APX) on the extracted phloem sap.

#### 3.4.1. Enzymatic Antioxidant Assays of SOD, CAT, DHAR and APX Enzymes

Catalase (CAT) activity was determined according to [65] with modifications. The mixture reaction consisted of 50 mM potassium phosphate buffer pH 7.2, 5 × 10^−3^ M H_2_O_2_ and 0.1 mL of phloem sap. The activity was monitored by H_2_O_2_ consumption and the consequent decrease in absorbance at OD 240 nm. One CAT unit is defined as the amount of enzyme necessary to decompose 1 µmol.min^−1^ of H_2_O_2_.

Superoxide dismutase (SOD) activity was measured using the method described in [66], which is based on the photo-reduction of nitroblue tetrazol (NBT). The addition of superoxide dismutase leads to an inhibition of the color reaction. The reactive mixture contained 1 mL of 25 µM riboflavin, 10 mM methionine, 50 mM nitroblue tetrazolium (NBT), 50 mM potassium phosphate buffer pH 7.8 and 1 µL of phloem sap. After 10 min of incubation at 250 μmol m^−2^ s^−1^ light, the absorbance was measured at 560 nm. This method is based on the inhibition of NBT photoreduction. The values were presented as the inverse of the concentration providing 50% inhibition (1/IC_50_).

Dehydroascorbate reductase (DHAR) activity was assayed by monitoring the increase in absorbance at OD 265 nm due to glutathione-dependent ascorbate production [67]. The 500 µL reactive mixture consisted of 0.1 M phosphate buffer pH 6.2, 2 mM reduced glutathione (GHS), 1 µL of phloem sap and 50 µL of 1 mM dehydroascorbate, the latter being responsible for the start of the reaction.

Ascorbate peroxidase (APX) activity was determined by using the method described by [68]. The 2.9 mL reaction mixture contained 0.25% (*v*/*v*) of guaiacol solubilized in sodium phosphate buffer 10 mM pH 6 and 100 mM H_2_O_2_. To initiate the reaction, 100 µL of phloem sap was added and the absorbance at 470 nm was measured after 10 min.

#### 3.4.2. Statistical Analysis

Statistical analysis of antioxidant activities was undertaken with a one-way ANOVA followed by a Tukey post-hoc test with the level of significance set for each treatment, using the statistics software Past 4.02 [68]. Comparisons were made between control and infected samples and between the infected samples. ****: *p* < 0.0001; ***: *p* < 0.001; **: *p* < 0.01; *: *p* < 0.05; n.s.: not significant (*p* > 0.05).

### 3.5. Analysis by UHPLC-HRMS and Data Processing

Metabolite profiles in the phloem sap of CTV infected and non-infected citrus plants CA and CM were studied by UHPLC-HRMS.

The chromatographic separation was achieved using a Thermo Scientific (Bremen, Germany) *ultimate* 3000 UHPLC (Bremen, Germany). The column was a Thermo Scientific Accucore RP-18 (2.1 × 100 mm, 2.6 µm). The mobile phase composition was prepared with water (A) and acetonitrile (B), both containing 0.1% of formic acid. The gradient (in *v*/*v* %) started with 100% of A for 2 min, increased linearly to 30% of B in 13 min, to 100% of B in 16 min, was maintained at 100 of % B for 4 min, returned to 100% of A in 1 min and then was maintained at 100% of A for 4 min before the next run. The flow rate was 0.3 mL/min. The injection volume was 5 µL of phloem sap diluted two times with MiliQ water.

Mass analysis was performed on an Orbitrap Elite (Bremen, Germany) mass spectrometer with a Heated EletroSpray Ionization source (HESI-II). Acquisition was performed under positive and negative polarities. HR-MS data were acquired using the following ionization parameters: spray voltages, 3.7 kV (positive polarity) and 4.0 kV (negative polarity); sheath gas, 40 arbitrary units; auxiliary gas, 10 arbitrary units; heater temperature, 300 °C; capillary temperature, 350 °C; S-Lenses RF level, 64.9%. Scan range was 100–1000 *m*/*z*. Fragmentation spectra were obtained by Collision Induced Dissociation (CID) by running the system in data dependent mode with dynamic exclusion. LC-MS profiles were analyzed using Compound Discoverer 3.3. Similarity of more than 85% (mzCloud database) and *p*-values < 0.001 or < 0.01 were employed as criteria for selecting marker compounds.

Three biological replicates of healthy CA (sensitive to CTV), healthy CM (tolerant to CTV), infected CA and infected CM were analyzed. The LC-HRMS^2^ profiles were analyzed and compounds annotated using Compound Discoverer 3.3 (Bremen, Germany), which has access to several online mass spectral databases, including mzCloud, (Bremen, Germany), Plantcyc and HMDB, and to the Arita’s lab Flavonoid Structure Database. The profiles were processed using an untargeted metabolomics workflow, which finds and annotates the differences between samples. In brief, it performs retention time alignment, unknown compound detection, and compound grouping across all samples, predicts elemental compositions for all compounds, fills gaps across all samples and performs similarity search for all compounds with ddMS2 data using mzCloud, applies mzLogic algorithm to rank order ChemSpider results and QC-based batch normalization, calculates differential analysis (*t*-test or ANOVA), determines *p*-values, adjusted *p*-values, ratios, fold change, etc. Different groups of individuals were compared, namely the ratios of the infected versus the non-infected groups for each species.

## 4. Conclusions

CA and CM have a distinct susceptibility to the severe CTV isolate T36, CA is a low susceptibility host, while CM is highly susceptible. Understanding CA and CM responses to severe CTV isolates is critical to understanding of the host-virus interaction and factors underpinning host susceptibility and to establishing new tools for virus control. In this study, a huge diversity and significant amounts of secondary metabolites were detected in the phloem sap of healthy CA, composed mainly of flavonoids. In contrast in healthy CM, phenolic acids were the more abundant secondary metabolites in phloem sap.

Upon infection by the quick decline T36 and the T318A stem pitting isolates, CA and CM had different responses. CM had a reaction similar to other plant species infected by viruses, although this differed between the two CTV isolates, with production of a significant number of compounds that confer pathogen resistance such as quercetin and lignan, pipecolic acid and SA affected to the SAR mechanism. CM infected with T36, but not T318A, had a high antioxidant enzyme activity and a significant increase in flavonoids and polymethoxylated flavonoids which may be related to increased H_2_O_2_ scavenging. CA metabolism suffered a drastic change upon infection by the quick decline isolate T36, with a reduced profile of secondary metabolites, including flavonoids and polymethoxylated flavonoids, and a low activity of CAT and SOD enzymes.

This study uncovers a complex CTV-host interaction, which depends on the CTV isolates and on the specific metabolism of the infected citrus plant. The main role attributed to flavonoids is the modulation of ROS, a natural defense system, so the decrease and alteration of these metabolites may result from a strong CTV-host interaction. We propose that the high concentration and diversity of flavonoid compounds in the phloem sap of healthy CA (Table 1), as well as the high activity of SOD and CAT, may explain its lower susceptibility to the T36 quick decline isolate. The metabolic response of CA seems to be particularly affected by severe CTV isolates, since both T36 and T318A affected the flavonoid biosynthesis and SOD and CAT antioxidant enzyme activity, and this may explain the susceptibility of different citrus species to viral infections. Our hypothesis is that flavonoid biosynthesis may be blocked by the virus. Future work needs to be developed to better understand the role of phenolic compounds in CTV infection and how they interfere with the CA response.

## Figures and Tables

**Table 1:** Metabolites tentatively annotated in the phloem sap of *Citrus aurantium* (CA) and *C. macrophylla* (CM). The ratios of annotated compounds, CA/CM, are listed in descending order. CA/CM ratio values below 0 indicate annotated metabolites are more abundant in CM. Listed compounds show mzCloud match ≥ 85% (negative or positive polarity) and *p*-value < 0.001. Compounds labelled with the symbol ^♦^ are also annotated in Table 2.

N°	Name	Formula	*m*/*z* (+/−)	mzCloud Match	Ratio CA/CM
1	^♦^ 5,3′-Dihydroxy-3,6,7,4′ tetramethoxyflavone	C_19_ H_18_ O_8_	373.0911 (−)	95.1	994
2	Eriodictyol	C_15_ H_12_ O_6_	289.0695 (+)	98.6	858
3	^♦^ Naringin	C_27_ H_32_ O_14_	579.1726 (−)	95.5	792
4	Rhoifolin	C_27_ H_30_ O_14_	579.1727 (+)	98.3	745
5	5,2′-Dihydroxy-6,7,8,6′ tetramethoxyflavone	C_19_ H_18_ O_8_	375.1062 (+)	91.1	419
6	5,7-Dihydroxy-6,3′,4′-trimethoxyflavone	C_18_ H_16_ O_7_	345.0953 (+)	95.0	204
7	Luteolin 7-rutinoside	C_27_ H_30_ O_15_	593.1530 (−)	91.5	152
8	^♦^ Quercetin	C_15_ H_10_ O_7_	303.0490 (+)	95.1	147
9	5,6,7,8,3′,4′-Hexamethoxyflavone	C_21_ H_22_ O_8_	403.1368 (+)	98.6	141
10	Apigenin	C_15_ H_10_ O_5_	271.0593 (+)	93.1	140
11	Naringenin	C_15_ H_12_ O_5_	273.0746 (+)	98.4	135
12	3-Methylkaempferol	C_16_ H_12_ O_6_	301.0700 (+)	99.3	132
15	Hesperetin	C_16_ H_14_ O_6_	303.0849 (+)	99.9	104
13	Neodiosmin	C_28_ H_32_ O_15_	609.1803 (+)	97.9	90
14	^♦^ Isoferulic acid	C_10_ H_10_ O_4_	195.0646 (+)	99.9	80
16	3-O-Feruloylquinic acid	C_17_ H_20_ O_9_	367.1019 (−)	95.5	84
17	Prunin	C_21_ H_22_ O_10_	435.1268 (+)	91.3	56
18	Isorhamnetin	C_16_ H_12_ O_7_	317.0647 (+)	97.4	51
19	^♦^ Neohesperidin	C_28_ H_34_ O_15_	609.1805 (−)	97.6	42
20	Apigenin 6,8-di-C-glucoside	C_27_ H_30_ O_15_	593.1532 (−)	93.0	40
21	Naringenin 4′-glucoside 7-rutinoside	C_33_ H_42_ O_19_	741.2214 (−)	100.0	32
22	^♦^ Biorobin	C_27_ H_30_ O_15_	593.1525 (−)	89.4	32
23	Vitexin	C_21_ H_20_ O_10_	423.1115 (+)	95.9	16
24	Methyl caffeate	C_10_ H_10_ O_4_	195.0646 (+)	98.3	9
25	4-Hydroxybenzaldehyde	C_7_ H_6_ O_2_	123.0436 (+)	99.4	0.50
26	Quercetin-3β-D-glucoside	C_21_ H_20_ O_12_	465.1017 (+)	98.2	0.34
27	Methyl cinnamate	C_10_ H_10_ O_2_	163.0755 (+)	96.7	0.25
28	4-Coumaric acid	C_9_ H_8_ O_3_	165.0540 (+)	99.8	0.18
29	3-[3-(beta-D-Glucopyranosyloxy)-2-methoxyphenyl] propanoic acid	C_16_ H_22_ O_9_	357.1173 (−)	96.9	0.13
30	Cnidioside A	C_17_ H_20_ O_9_	367.1019 (−)	99.6	0.09
31	Dihydrocaffeic acid	C_9_ H_10_ O_4_	181.0502 (-)	90.7	0.08
32	Asparagin	C_4_ H_8_ N_2_ O_3_	133.0603 (+)	96.1	0.07
33	Scopoletin	C_10_ H_8_ O_4_	193.0487 (+)	92.6	0.06
34	2,4-Dimethylbenzaldehyde	C_9_ H_10_ O	135.0799 (+)	87.2	0.06
35	7-Hydroxycoumarine	C_9_ H_6_ O_3_	163.0385 (+)	98.4	0.05
36	^♦^ 4-Aminobenzoic acid	C_7_ H_7_ N O_2_	136.0402 (−)	97.3	0.05
37	^♦^ Rutin	C_27_ H_30_ O_16_	609.1442 (−)	98.8	0.02
38	^♦^ Ferulic acid	C_10_ H_10_ O_4_	193.0509 (−)	99.1	0.01
39	L-(-)-Methionine	C_5_ H_11_ N O_2_ S	150.0671 (+)	99.8	0.007
40	Eriocitrin	C_27_ H_32_ O_15_	595.1650 (−)	99.5	0.006
41	^♦^ 2-Hydroxycinnamic acid	C_9_ H_8_ O_3_	163.0398 (−)	97.1	0.003

**Table 2:** Metabolites tentatively annotated in the phloem sap of infected and non-infected *Citrus aurantium* (CA) and *C. macrophylla* (CM). The ratios of annotated compounds are listed in descending order. Listed compounds have a mzCloud match ≥ 85% (negative or positive polarity) and a *p*-value < 0.01. Compounds labelled with the symbol ^♦^ were also annotated in Table 1.

**N°**	**Name**	**Formula**	***m*** **/** ***z***	**mzCloud Match**	**Ratio INF_CA T36/N-INF_CA**
1	3-[4-(beta-D-Glucopyranosyloxy)-6-methoxy-1-benzofuran-5-yl]propanoic acid	C_17_ H_20_ O_9_	367.1022 (−)	99.5	43
2	^♦^ Biorobin	C_27_ H_30_ O_15_	583.1520 (−)	89.4	0.147
**N°**	**Name**	**Formula**	***m*/*z***	**mzCloud Match**	**Ratio INF_CM T36/N-INF_CM**
1	2-[[6-hydroxy-4-(4-hydroxy-3,5-dimethoxyphenyl)-3-(hydroxymethyl)-5,7-dimethoxy-1,2,3,4-tetrahydronaphthalen-2-yl]methoxy]-6-(hydroxymethyl)oxane-3,4,5-triol	C_28_ H_38_ O_13_	581.2223 (−)	98.1	200
2	5,6′-Dihydroxy-6,7,8,2′-tetramethoxyflavone	C_19_ H_18_ O_8_	375.1061 (+)	91.1	16
3	Isovanillic acid	C_8_ H_8_ O_4_	169.0489 (+)	99.4	12
4	cis-Aconitic acid	C_6_ H_6_ O_6_	173.0086 (−)	89.2	9
5	Nobiletin	C_21_ H_22_ O_8_	403.1378 (+)	95.6	6
6	3,4-Dihydroxybenzaldehyde	C_7_ H_6_ O_3_	139.0387 (+)	97.4	5
7	^♦^ Quercetin	C_15_ H_10_ O_7_	303.0486 (+)	95.1	5
8	^♦^ Naringin	C_27_ H_32_ O_14_	579.1729 (−)	95.5	5
9	^♦^ 4-Aminobenzoic acid	C_7_ H_7_ N O_2_	136.0402 (−)	97.2	0.2
10	^♦^ Isoferulic acid	C_10_ H_10_ O_4_	195.0648 (+)	94.7	0.07
11	Glucose-1,6-bisphosphate	C_6_ H_14_ O_12_ P_2_	338.9875 (−)	99.1	0.03
12	Guanosine	C_10_ H_13_ N_5_ O_5_	338.9875 (−)	99.9	0.003
**N°**	**Name**	**Formula**	***m*/*z***	**mzCloud Match**	**Ratio INF_CA_T318A/N-INF_CA**
1	5,3′-Dihydroxy-3,6,7,4′-tetramethoxyflavone	C_19_ H_18_ O_8_	373.0994 (−)	94.4	403
2	Fumaric acid	C_4_ H_4_ O_4_	115.0035 (−)	99.5	55
3	cis-Aconitic acid	C_6_ H_6_ O_6_	173.0085 (−)	89.2	17
4	Isovanillic acid	C_8_ H_8_ O_4_	167.0346 (−)	96.6	0.4
5	3-[2-(β-D-Glucopyranosyloxy)-4-methoxyphenyl]propanoic acid	C_16_ H_22_ O_9_	357.1173 (−)	99.6	0.4
6	^♦^ 2-Hydroxycinnamic acid	C_9_ H_8_ O_3_	163.0398 (−)	91.5	0.18
7	^♦^ Neohesperidin	C_28_ H_34_ O_15_	609.1805 (−)	97.6	0.17
8	Hesperidin	C_28_ H_34_ O_15_	609.1825 (−)	98.5	0.002
**N°**	**Name**	**Formula**	***m*/*z***	**mzCloud Match**	**Ratio INF_CM_T318A/N-INF_CM**
1	3-[2-(β-D-Glucopyranosyloxy)-4-methoxyphenyl]propanoic acid	C_16_ H_22_ O_9_	357.1166 (−)	99.1	1977
2	Pipecolic acid	C_6_ H_11_ N O_2_	130.0862 (+)	98.1	109
3	Salicylic acid	C_7_ H_6_ O_3_	137.0233 (−)	99.8	22
4	Proline	C_5_ H_9_ N O_2_	116.0704 (+)	100.0	21
5	3,4-Dihydroxyphenylpropionic acid	C_9_ H_10_ O_4_	181.0509 (−)	90.7	0.023
6	Hesperidin	C_28_ H_34_ O_15_	609.1824 (−)	98.5	0.019
7	^♦^ Ferulic acid	C_10_ H_10_ O_4_	193.0513 (−)	98.9	0.013
8	^♦^ 4-Aminobenzoic acid	C_7_ H_7_ N O_2_	136.0402 (−)	97.3	0.011
9	Isovanillic acid	C_8_ H_8_ O_4_	167.0345 (−)	96.1	0.008
10	Guanosine	C_10_ H_13_ N_5_ O_5_	338.9874 (−)	99.9	0.005
11	^♦^ Rutin	C_27_ H_30_ O_16_	609.1443 (−)	98.8	0.005

## Data Availability

All data included in the main text.
